# Expression of homologous RND efflux pump genes is dependent upon AcrB expression: implications for efflux and virulence inhibitor design

**DOI:** 10.1093/jac/dku380

**Published:** 2014-10-06

**Authors:** Jessica M. A. Blair, Helen E. Smith, Vito Ricci, Amelia J. Lawler, Louisa J. Thompson, Laura J. V. Piddock

**Affiliations:** Antimicrobials Research Group, School of Immunity and Infection, College of Medical and Dental Sciences, and Institute of Microbiology and Infection, The University of Birmingham, Birmingham B15 2TT, UK

**Keywords:** redundancy, AcrB, AcrD, AcrF

## Abstract

**Objectives:**

Enterobacteriaceae have multiple efflux pumps that confer intrinsic resistance to antibiotics. AcrB mediates clinically relevant multidrug resistance and is required for virulence and biofilm formation, making it an attractive target for the design of inhibitors. The aim of this study was to assess the viability of single transporters as a target for efflux inhibition using *Salmonella* Typhimurium as the model pathogen.

**Methods:**

The expression of resistance–nodulation–division (RND) efflux pump genes in response to the inactivation of single or multiple homologues was measured using real-time RT–PCR. Phenotypes of mutants were characterized by measuring antimicrobial susceptibility, dye accumulation and the ability to cause infection *in vitro*.

**Results:**

The expression of all RND efflux pump genes was increased when single or multiple *acr* genes were inactivated, suggesting a feedback mechanism that activates the transcription of homologous efflux pump genes. When two or three *acr* genes were inactivated, the mutants had further reduced efflux, altered susceptibility to antimicrobials (including increased susceptibility to some, but conversely and counterintuitively, decreased susceptibility to some others) and were more attenuated in the tissue culture model than mutants lacking single pumps were.

**Conclusions:**

These data indicate that it is critical to understand which pumps an inhibitor is active against and the effect of this on the expression of homologous systems. For some antimicrobials, an inhibitor with activity against multiple pumps will have a greater impact on susceptibility, but an unintended consequence of this may be decreased susceptibility to other drugs, such as aminoglycosides.

## Introduction

Efflux is an important mechanism of multidrug resistance in bacteria, conferring decreased susceptibility to a wide range of substrates including antibiotics, dyes, detergents and biocides.^1^ This makes them an attractive target for the design of inhibitors that could be used to potentiate the use of existing antimicrobials.

Resistance–nodulation–division (RND) efflux transporters are found in the inner membrane of Gram-negative bacteria and form a complex with an outer membrane channel and a periplasmic adaptor protein (PAP) to form a tripartite efflux pump system spanning both the inner and outer membrane.^2,3^ The substrates of these multiprotein complexes are structurally diverse and include antibiotics, biocides, dyes, detergents and host-derived molecules. The active efflux of substrates by RND systems is responsible for the intrinsic resistance of Gram-negative bacteria to multiple classes of structurally distinct antimicrobials.^1^

*Salmonella* have five RND efflux pump systems: AcrAB, AcrD, AcrEF, MdtABC and MdsABC. Further RND efflux pumps are found in some other members of the Enterobacteriaceae, including MdtF in *Escherichia coli.*^4^ The transporter protein AcrB, and its homologues in other Gram-negative bacteria, is considered the most clinically relevant RND system because it has the broadest substrate profile and is more abundant within the cell than are other efflux systems.^1^ Inactivation of *acrB* increases the susceptibility of laboratory mutants of *E. coli*, *Salmonella enterica* and other Enterobacteriaceae to many antimicrobials, whereas overexpression confers multidrug resistance, including to clinically efficacious agents. Such mutants have been selected *in vitro* and *in vivo.*^5–8^ Efflux via AcrB is driven by the proton-motive force and forms a tripartite complex with PAP, AcrA and the outer membrane channel TolC.^9^ The recent elucidation of the structure of AcrB in complex with different substrates of varying molecular weights has revealed two large, discrete, multisite binding pockets within AcrB, which may explain how AcrB can transport such structurally varied substrates.^10,11^

Single deletions of RND efflux pump genes other than *acrB* have little or no effect on the susceptibility of Enterobacteriaceae to most antimicrobial agents.^12^ The antimicrobial susceptibility of deletion mutants and strains with an increased expression of certain RND efflux pumps indicates that there is an overlap or redundancy between the antimicrobials, biocides, dyes and detergents that can be transported by the different RND pumps of *Salmonella.*^12,13^ AcrF is the closest homologue of AcrB in *Salmonella* (80% identity) and AcrEF overexpression can suppress antibiotic hypersusceptibility in AcrB-deficient strains.^12,13^ AcrD (64% identity to AcrB) and MdtABC have similar substrate profiles including SDS, novobiocin, deoxycholate, some β-lactams, copper and zinc.^12,14^ In *E. coli* and *Salmonella*, AcrD also transports aminoglycoside antibiotics.^15,16^ MdsABC is found only in *Salmonella* and in LB medium is expressed at lower levels than the other four RND efflux pumps.^17^ However, in a strain lacking AcrB and overexpressing *mdsAB*, encoding the pump and PAP, or *mdsABC*, encoding all three components, susceptibility to novobiocin, acriflavine, Crystal Violet, methylene blue, rhodamine 6G, benzalkonium chloride and SDS was decreased compared with the AcrB mutant alone. This suggests that MdsB is capable of exporting these compounds.^18^

In addition to their role in resistance to antimicrobials, some RND efflux pumps are also required for the virulence of many Gram-negative pathogens.^19^ In *Salmonella* Typhimurium, inactivation of *acrB* attenuated the invasion of tissue culture cells *in vitro* and colonization in poultry.^20,21^ Inactivation of *acrAB* or *acrEF* has also been shown to attenuate the virulence of the organism in mice.^12^

The regulation of RND efflux pumps is complex; transcriptional control is multilayered and some regulators control the expression of more than one pump. In *Salmonella* and *E. coli*, the regulation of *acrAB* is the best studied. The *acrA* and *acrB* genes are encoded in a single operon and are co-regulated. At a local level, *acrAB* is repressed by AcrR, which is encoded alongside and divergently transcribed from *acrAB*. At a global level, members of the AraC/XylS family of DNA transcriptional activators, such as MarA, SoxS, Rob and RamA, all influence the expression of *acrAB-tolC* and have overlapping recognition sites.^22–27^ AcrD and MdtABC are both under the control of the two-component regulatory systems BaeSR and CpxAR, which induce the expression of AcrD and MdtABC in response to high levels of indole, copper, zinc or envelope stress.^28,29^ The expression of *acrEF* in *E. coli* is generally low due to repression by the global regulator H-NS.^30^ However, *acrEF* is also encoded alongside a gene encoding a local repressor, AcrS (previously EnvR), which inhibits the expression of *acrAB* and acts as a regulatory switch between the expression of *acrAB* and *acrEF*.^31^

Due to the functional redundancy of RND pumps, potential exists for the loss of certain pump components to be compensated by increased expression of a homologous component that could fulfil, at least to some extent, the same function. For example, Eaves *et al*.^32^ showed that when the *acrB* or *acrF* of *Salmonella* was inactivated, the expression of *acrD* increased (3.6-fold and 4.9-fold, respectively). To allow compensatory changes in the expression levels of efflux systems upon the inactivation of homologous systems, there must be a tightly controlled and integrated regulatory network that can respond to a loss of efflux function. While the literature contains multiple examples of the regulation of single efflux systems or a small number of efflux systems, an integrated network of regulation is yet to be elucidated.

The role of RND systems in both antimicrobial resistance and virulence makes them attractive targets for the design of inhibitors. Using *Salmonella* Typhimurium as a model, the aim of the present study was to investigate the viability of single transporters such as AcrB as a target for efflux inhibition by investigating the expression and roles of structurally similar efflux pumps in antimicrobial resistance and virulence, as well as the extent of the redundancy between RND efflux pumps. This information is crucial for the rational design of inhibitors that inhibit all pumps, thus preventing resistance by a compensatory overproduction of homologous RND efflux systems.

## Materials and methods

### Strains and growth conditions

All strains were derived from *S. enterica* serovar Typhimurium SL1344.^33^ *Salmonella* was used as a model organism in this study as it is an important human pathogen that causes a significant number of infections annually. SL1344 is a widely studied pathogenic strain of *Salmonella* for which there are well-validated models of infection including an *in vitro* tissue culture model. Single-gene inactivated mutants were constructed as previously described.^32,34^ Mutants with multiple inactivated efflux pumps were created by P22 transduction between mutants in which single genes were inactivated or deleted. All mutants were verified by PCR and DNA sequencing. All experiments including MICs reveal that the phenotype of the marked and unmarked mutants for the same gene is indistinguishable. LB broth (Sigma-Aldrich, UK) and MOPS minimal medium (Teknova Inc., USA) were used throughout this study.

### RNA extraction and real-time quantitative RT–PCR

Overnight cultures of *Salmonella* Typhimurium SL1344 and the test strains were grown in MOPS minimal medium at 37°C. From each strain, three biological replicate RNA preparations were made and quantified as previously described.^22,35^ Primers (Table S1, available as Supplementary data at *JAC* Online) were designed with an annealing temperature of 57.3°C using Beacon Designer 4.0 (Premier Biosoft, USA). cDNA was synthesized from 2 μg of total RNA using the SuperScript III cDNA synthesis kit (Invitrogen). Validation experiments were carried out using five cDNA standards of different concentrations (10, 1, 0.1, 0.01 and 0.001 ng/μL) to determine PCR efficiency for the housekeeping gene 16S and each test gene. Quantitative RT–PCRs were set up in biological triplicate and technical duplicate in a Bio-Rad PCR tray using 1 μL of neat cDNA for the test genes and 1 μL of a 1: 1000 dilution of cDNA for 16S in a 25 μL reaction containing 12.5 μL of iQ SYBR Green Supermix (Bio-Rad, UK), 1 μL of primers (500 nM) and 9.5 μL of sterile water. Quantitative RT–PCR was carried out in a CFX96 real-time machine (Bio-Rad, UK) using the following protocol: 95°C for 5 min followed by 40 plate read cycles of 95°C for 30 s, 57.3°C for 30 s and 72°C for 30 s. Data were analysed using CFX Manager (Bio-Rad, UK) and expression ratios were calculated using the ΔΔCt method and normalized to the expression of 16S.^36^

### Determination of susceptibility to antimicrobials

Biolog Phenotype Microarray data were confirmed by measuring growth in the presence of representative AcrB substrates. Briefly, overnight bacterial cultures were diluted to 10^4^ cfu/mL and grown in a 96-well plate in Iso-Sensitest broth in the presence of selected drugs at 0.25× the WT MIC.

The MICs of antibiotics, dyes and detergents were determined for each strain according to the standardized agar doubling dilution method procedure of the BSAC using Iso-Sensitest agar.^37^ The MIC was determined as the lowest concentration of antimicrobial that caused no visible growth. The values stated are the mode value from at least three biological replicates performed on at least three independent occasions. All the antimicrobials tested were obtained from Sigma-Aldrich, UK.

### Accumulation of Hoechst 33342 and norfloxacin

The efflux activity of the mutants was assessed by determining the accumulation of the fluorescent dye Hoechst 33342 and norfloxacin (Sigma-Aldrich, UK) as previously described.^38,39^ Differences in steady-state accumulation values between mutants and parental strains were analysed for statistical significance using a two-tailed Student's *t*-test, where *P*<0.05 was considered significant. Data presented are the means of three independent biological replicates (±SEM).

### Adhesion and invasion assays

The ability of the strains to adhere to, and invade, INT 407 (human embryonic intestine cells) was measured as previously described.^21^ Each assay was repeated a minimum of three times, with each experiment including four technical replicates per bacterial strain. The results were analysed using Student's *t*-test and *P* values of ≤0.05 were considered significant.

## Results

### In silico analysis

The *Salmonella* genome encodes five efflux systems of the RND family. The three most similar pump proteins are AcrB, AcrD and AcrF (Table 1). The two remaining RND pumps, MdsB and MdtB/C, which is a heteromultimer of MdtB and MdtC, are 63.4% and 46.7%/48.6% identical to AcrB, respectively. ClustalW alignments of all six RND type proteins revealed that five residues (Asp407, Asp408, Lys940, Arg971 and Thr978) vital to proton transport within the AcrB of *E. coli*^40,41^ are identical in all six *Salmonella* proteins, indicating that energy transduction is conserved in this family of proteins. However, greater variation was seen in residues involved in substrate recognition or binding. For example, of six important residues in the hydrophobic, phenylalanine-rich binding pocket of AcrB (Phe178, Phe615, Phe610, Phe136, Phe617 and Phe628),^42^ all six were conserved in AcrF, two in AcrD, one in MdtC, one in MdsB and none of these residues in MdtB.

**Table 1.** Percentage nucleotide identity and amino acid similarity between the RND efflux pump genes and proteins of *Salmonella*

	*acrB/*AcrB	*acrD/*AcrD	*acrF/*AcrF	*mdtB/*MdtB	*mdtC*/MdtC	*mdsB/*MdsB
*acrB*/AcrB	—	70/79.1	74/90.4	55/46.7	55/48.6	59/63.4
*acrD*/AcrD	—	—	68/78.2	54/49.1	54/48.8	59/61.3
*acrF*/AcrF	—	—	—	54/47.4	53/48.5	57/63.1
*mdtB*/MdtB	—	—	—	—	62/66.0	56/49.0
*mdtC*/MdtC	—	—	—	—	—	56/49.2
*mdsB*/MdsB	—	—	—	—	—	—

### Expression of efflux pump genes is altered upon inactivation of homologous pumps

To determine whether the expression of each gene was altered upon inactivation of one or more homologous genes, real-time quantitative RT–PCR was used to measure the level of *acrB*, *acrD* and *acrF* transcription in the single, double and triple efflux mutants compared with SL1344 (Table 2). As previously reported,^32^ in the *acrB* mutant, both *acrD* and *acrF* showed increased expression. In the *acrD* mutant, the expression of *acrB* was increased while *acrF* expression was unchanged. In the *acrF* mutant, both *acrB* and *acrD* showed increased expression. The expression of *mdsB* and *mdtB* was also affected by the loss of single RND pumps. The expression of *mdtB* was increased in the *acrF* mutant while expression of the *Salmonella*-specific efflux pump *mdsB* was decreased upon inactivation of *acrB*, *acrD* or *acrF* (Table 2).

**Table 2.** Expression of RND efflux pump genes and their regulators, quantified by real-time RT–PCR

Strain		Fold change in gene expression										
		RND efflux pump genes					known regulators of efflux					
		*acrB*	*acrD*	*acrF*	*mdtB*	*mdsB*	*marA*	*ramA*	*rob*	*soxS*	*acrR*	*envR*
SL1344	WT	1.0	1.00	1.0	1.0	1.0	1.0	1.0	1.0	1.0	1.0	1.0
L644	Δ*acrB*	—	**13.4**	**16.0**	1.0	*0*.*6*	**2.0**	**2.8**	1.4	**2.6**	**0.4**	1.2
L132	*acrD::aph*	**1.8**	—	1.0	0.9	*0*.*5*	1.2	0.8	1.4	1.3	0.3	0.6
L131	*acrF::aph*	**3.4**	**3.4**	—	**1.8**	*0*.*7*	0.9	1.0	1.3	1.4	0.9	1.5
L646	Δ*acrF acrB::aph*	—	**4.6**	—	**4.5**	**3.0**	**2.5**	1.6	1.2	**6.3**	0.4	0.8
L1297	Δ*acrB acrD::aph*	—	—	**2.4**	**4.6**	**2.1**	1.2	1.4	1.1	1.8	2.2	1.2
L1395	*acrD::cat acrF::aph*	**3.8**	—	—	**6.0**	**5.9**	1.0	1.3	1.4	**2.2**	0.4	1.0
L1405	Δ*acrB* Δ*acrF acrD::aph*	—	—	—	1.1	**3.2**	1.6	1.9	1.0	**5.0**	0.5	**4.5**

Bold font indicates statistically significant (*P *≤ 0.05) increased expression. Italic font indicates statistically significant (*P *≤ 0.05) decreased expression.

When two genes were inactivated (e.g. *acrB* and *acrF* or *acrB* and *acrD*), the expression of *acrD* or *acrF* was increased, although expression was lower than in the strain lacking only *acrB* (L110). When *acrF* and *acrD* were inactivated, the expression of *acrB* was increased; this was greater than that seen upon inactivation of *acrD* and similar to that in the *acrF* mutant. Expression of the *mdtB* and *mdsB* efflux genes was increased in all *acr* gene double mutants (L646, L1297 and L1395), but in the triple *acrBDF* mutant only *mdsB* expression was increased. The expression of both *mdtB* and *mdsB* was highest in the *acrDF* mutant (L1395) (Table 2).

The expression level of known regulators of RND efflux was also measured. The expression of *ramA* and *marA* was increased when *acrB* was inactivated but was not changed in the *acrD* (L132) or *acrF* (L131) mutants (Table 2). The expression of *soxS* was increased in the *acrBF* mutant (6.3-fold), in the *acrDF* mutant (2.2-fold) and in the strain lacking all three RND pump genes. The expression of *rob* was not significantly altered in any of the mutants. The expression of the genes encoding the repressor proteins AcrR and AcrS was also measured. The transcription of *acrR* was decreased in the *acrB* mutant and transcription of *acrS* was increased in the *acrBDF* mutant (Table 2).

### Inactivation of two or more RND efflux systems altered antimicrobial susceptibility

**Figure 1.**
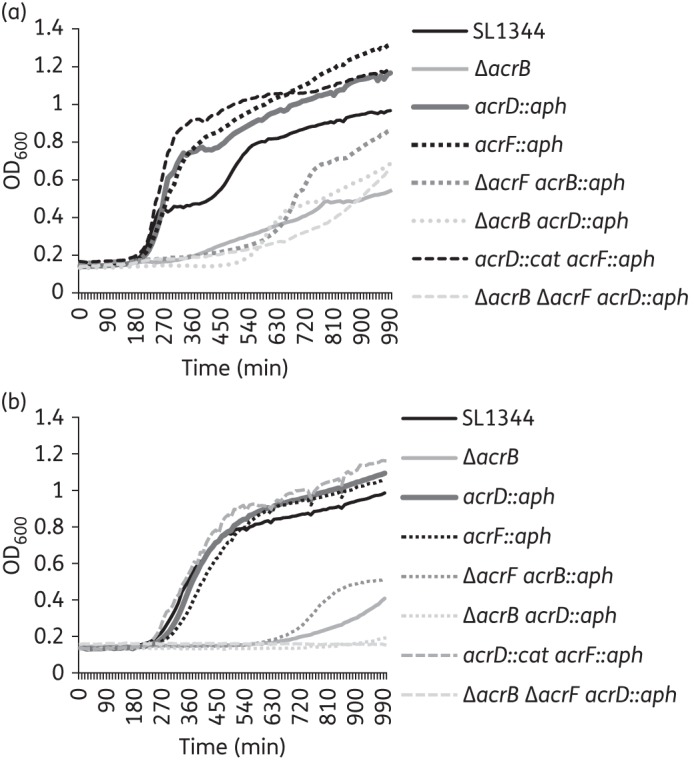
Growth of efflux pump mutants in the presence of AcrB substrates. Growth of the efflux pump mutants in the presence of (a) ciprofloxacin and (b) doxycycline at a concentration of 0.25× the MIC for the WT. The data presented are the means of three biological replicates.

As previously described, the inactivation of *acrB* led to multidrug hypersusceptibility while the single inactivation of either *acrD* or *acrF* did not significantly alter the MICs of antibiotics, dyes and detergents compared with the WT strain. A reinterrogation of previously published data^43^ from the Biolog Phenotype Microarray showed that the *acrD* and *acrF* mutants grew better than SL1344 when exposed to four β-lactams, five macrolides and five quinolones (Table S2). This observation was confirmed by measuring the growth kinetics of the strains in the presence of representative AcrB substrates. However, the beneficial effect of lacking *acrD* or *acrF* was lost when *acrB* was deleted in the same strain (L1297 and L646, respectively) (Figure 1).

The antimicrobial susceptibility of the double mutant lacking AcrD and AcrF (L1395) was not significantly different from that of SL1344 (Table 3). Furthermore, except for ethidium bromide (for which the MIC value was lower) and the aminoglycosides (for which the MIC values were increased), the susceptibility of the *acrBD* (L1297), *acrBF* (L646) and the triple *acrBDF* (L1405) mutants to antibiotics, dyes and detergents was not significantly different from that of the *acrB* mutant. Surprisingly, the MICs of the aminoglycoside antibiotics, streptomycin, gentamicin and amikacin, were higher for the *acrBF* (L646) mutant and the triple *acrBDF* mutant (L1405) than for the WT parental strain SL1344; the MIC of tobramycin was also greater for L1405 than SL1344 (Tables 3 and 4). All *acrB* mutants (L110, L646, L1297 and L1405) were more susceptible to the efflux inhibitors phenylalanine-arginine β-naphthylamide (PABN) and carbonyl cyanide *m*-chlorophenylhydrazone (CCCP) than the WT parental strain (SL1344).

**Table 3.** MICs of antimicrobials for SL1344 and its mutants

	Genotype	MIC (mg/L)													
		AMP	CHL	CIP	TET	NAL	EtBr	FUS	AMK	GENT	HYG	STR	TOB	PABN	CCCP
SL1344	WT	2	4	0.015	1	4	>256	>256	4	0.5	32	8	2	>1024	64
L110	*acrB::aph*	*0.25*	*1*	<0.008	0.5	*1*	*64*	*8*	4	1	32	8	1	*64*	32
L644	Δ*acrB*	*0.25*	*1*	<0.008	0.5	*1*	*64*	*8*	4	1	32	8	1	*64*	32
L131	*acrF::aph*	2	4	0.015	2	4	>256	>256	4	1	32	16	2	>1024	64
L132	*acrD::aph*	2	4	0.015	1	4	>256	>256	4	1	32	16	2	>1024	64
L646	Δ*acrF acrB::aph*	2	*1*	<0.008	1	*1*	*16*	*4*	8	**2**	64	**32**	4	*64*	32
L1297	Δ*acrB acrD::aph*	*0.25*	*1*	<0.008	0.5	*1*	*64*	*8*	2	0.5	32	8	1	*64*	32
L1395	*acrD::cat acrF::aph*	2	16	0.015	2	4	>256	>256	4	1	64	16	2	>1024	64
L1405	Δ*acrB* Δ*acrF acrD::aph*	*0.12*	*1*	<0.008	0.5	*1*	*16*	*8*	**16**	**2**	64	**32**	**8**	*64*	32

AMP, ampicillin; CHL, chloramphenicol; CIP, ciprofloxacin; TET, tetracycline; NAL, nalidixic acid; EtBr, ethidium bromide; FUS, fusidic acid; AMK, amikacin; GEN, gentamicin; HYG, hygromycin; STR, streptomycin; TOB, tobramycin.

The *aph* gene used is *aph*(3′)-1, which provides resistance to kanamycin, neomycin and paromomycin.

Bold font indicates an increase in the MIC of the same compound compared with SL1344. Italic font indicates a decrease in the MIC of the same compound compared with SL1344.

**Table 4.** Fold change in MIC compared with Δ*acrB* (L644)

	Genotype	AMP	CHL	CIP	TET	NAL	EtBr	FUS	AMK	GEN	HYG	STR	TOB	PABN	CCCP
L646	Δ*acrF acrB::aph*	**4**			**2**		*−4*	*−2*	**2**	**2**	**2**	**4**	**4**		
L1297	Δ*acrB acrD::aph*								*−2*	*−2*	*−2*				
L1405	Δ*acrB* Δ*acrF acrD::aph*	*−2*					*−4*		**4**	**2**	**2**	**4**	**8**		

AMP, ampicillin; CHL, chloramphenicol; CIP, ciprofloxacin; TET, tetracycline; NAL, nalidixic acid; EtBr, ethidium bromide; FUS, fusidic acid; AMK, amikacin; GEN, gentamicin; HYG, hygromycin; STR, streptomycin; TOB, tobramycin.

The *aph* gene used is *aph*(3′)-1, which gives resistance to kanamycin, neomycin and paromomycin.

Bold font indicates an increase in MIC compared with the same compound for L644. Italic font indicates a decrease in the MIC of the same compound for Δ*acrB*. No value indicates no difference in MIC values.

### Inactivation of two or more RND efflux systems decreased efflux activity

**Figure 2.**
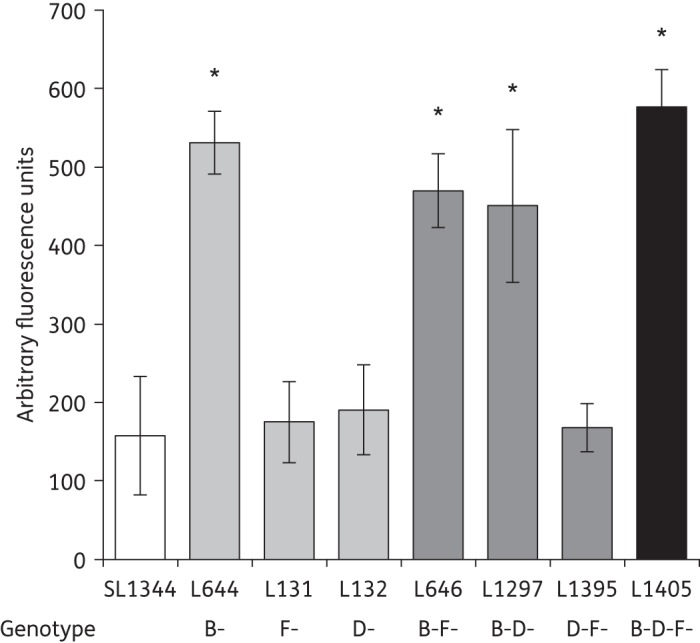
Hoechst 33342 accumulation in single, double and triple efflux mutants. The data presented are the means of three separate experiments presented as fold changes compared with SL1344 at the endpoint of the assay (±SEM). Student's *t*-tests were performed to compare the accumulation of Hoechst 33342 by each strain with that of SL1344. Those returning *P* values of <0.05 are indicated by asterisks.

**Figure 3.**
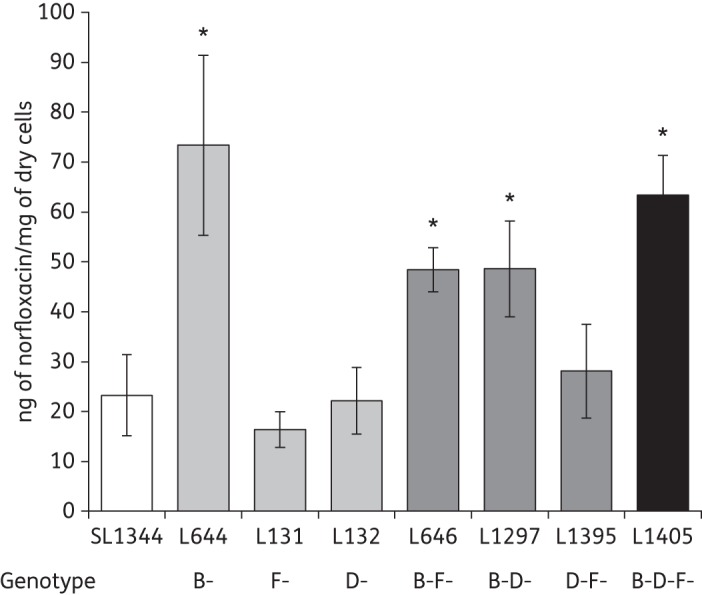
Accumulation of norfloxacin. The data shown are the means of three biological replicates (±SEM). Student's *t*-tests were performed to compare the accumulation of norfloxacin by each strain with that of SL1344. Those returning *P* values of <0.05 are indicated by asterisks.

We previously showed that the inactivation of *acrB* led to an increased accumulation of the dye Hoechst 33342.^20^ Compared with SL1344, the inactivation of *acrD* (L132) or *acrF* (L131) or the inactivation of both *acrD* and *acrF* (L1395) did not significantly alter the accumulation of Hoechst 33342 (Figure 2). However, mutants with inactivation of *acrB* and another *acr* gene [*acrBD* (L1297) or *acrBF* (L646)] accumulated less Hoechst 33342 than the *acrB* mutant. The *acrBDF* mutant (L1405) accumulated the highest level of Hoechst 33342, indicating the lowest level of efflux. Accumulation of the fluoroquinolone antibiotic norfloxacin showed a similar pattern, although the *acrB* mutant accumulated a higher concentration than the *acrBDF* mutant (Figure 3).

### Inactivation of two or more RND efflux systems attenuated the ability of Salmonella to infect tissue culture cells

**Figure 4.**
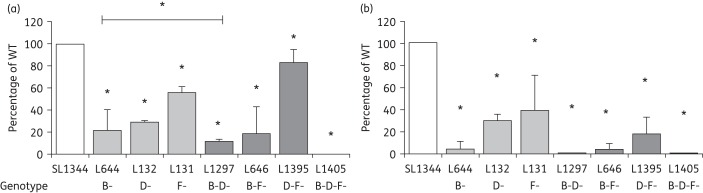
Adhesion to (a) and invasion of (b) INT 407 cells *in vitro* by strains lacking AcrB, AcrD, AcrF and combinations of these. The data shown are the means of at least three independent experiments. Student's *t*-tests were used to compare the values for each strain with that of the WT, SL1344. *P* values of ≤0.05 were considered significant and are indicated by asterisks.

In addition to their role in antimicrobial resistance, in many Gram-negative bacterial pathogens RND efflux pumps are required for the ability to cause infection.^19^ Inactivation of *acrB* has been previously shown to attenuate the invasion of *Salmonella* Typhimurium into mammalian cells growing in tissue culture.^12,20,21^ We now show that single-gene inactivation of either *acrD* (L132) or *acrF* (L131) also significantly attenuates the virulence of *Salmonella*. Adhesion of L131 (*acrF::aph*) to human intestinal cells (INT 407) was 56.0% that of SL1344 and it invaded at only 39.1% of the WT level. L132 (*acrD::aph*) was even more attenuated; adhesion being only 29.0% that of SL1344 and invasion only 39.1% of the SL1344 level (Figure 4).

When two efflux pump genes were inactivated or deleted, the ability of *Salmonella* to adhere to, and invade, INT 407 cells was attenuated more than that seen in single *acr* mutants (Figure 4). Invasion of the *acrBD* double mutant was the lowest among all single and double mutants, at only 0.4% of the WT level. The adhesion of the *acrDF* mutant, L1395 (*acrD::cat*, *acrF::aph*), was 83.7% that of the adhesion of SL1344, which is significantly greater than that of mutants lacking only one of these systems. Despite this, the invasion level of this mutant was only 17.8% that of the parental strain, showing that these two mutations have an additive effect upon invasive ability. When all three of the efflux genes were inactivated, in L1405, *Salmonella* was almost completely unable to adhere to or invade INT 407 cells (adhesion = 0.16% of the WT level, invasion = 0.004% of the WT level).

## Discussion

The role of RND efflux pumps in multidrug resistance and virulence makes them attractive targets for the design of efflux inhibitors. We have shown that the expression of all RND efflux pump genes can be altered when single or multiple *acr* genes are inactivated. These data suggest that the bacterium can sense and respond to the levels of RND transporters and, due to overlapping substrate specificity, this affords the bacterium resilience to prevent the intracellular accumulation of toxic metabolites,^44–47^ or to increase survival in toxic environments.

Critically, we can correlate alterations in the efflux level and sensitivity to antimicrobials to the compensatory changes in efflux pump gene expression in strains lacking specific RND pumps. For example, in the Biolog Phenotype Microarray many of the compounds in which the *acrD* and *acrF* mutant grew better are known substrates of AcrB, which is overexpressed in these mutants. Furthermore, this beneficial effect is lost when *acrB* is also inactivated. Other than to the aminoglycosides, the susceptibility of the *acrBD* and *acrBF* mutants was not significantly different from that of the mutant lacking only *acrB* and it is likely that this is because the other three RND systems are overproduced in both cases.

AcrD, which is known to transport aminoglycosides, and MdtB, which has a similar substrate profile to AcrD, are overexpressed in the *acrBF* mutant. This could explain the decreased susceptibility to the aminoglycosides seen in this mutant. The *acrBDF* mutant also had decreased susceptibility to the aminoglycosides. The expression of *mdsB* is increased in this mutant but there is currently no evidence that this pump can transport aminoglycosides. Aminoglycosides enter bacterial cells by self-promoted uptake and it is possible that changes in the expression of genes encoding cell envelope components, including LPS, could be responsible for this effect.^48^

These data provide proof of principle that changes in the expression of pumps in response to the inactivation of RND efflux pumps can alter the susceptibility to clinically relevant antimicrobials. We postulate the same will be true when the pump proteins themselves are inhibited, and recent evidence showing that the efflux inhibitors PABN and 1-(1-naphthylmethyl)-piperazine (NMP) altered the expression of RND efflux pump genes in *E. coli* supports this.^49^ Additionally, this highlights the fact that any change in the phenotype of strains with single or multiple genes inactivated should be interpreted with caution as the phenotype represents the engineered inactivation and any consequent transcriptional changes.

The role of AcrAB-TolC in the virulence of *Salmonella* Typhimurium is well established and inactivation of *acrB* causes a decreased expression of genes in *Salmonella* Pathogenicity Island (SPI-1), which are known to be required for infection.^12,20,21,35^ Nishino *et al*.^12^ showed that the inactivation of *acrD* did not confer significant attenuation in the BALB/C mouse model of infection and the inactivation of *acrEF* (encoding the RND pump protein and the PAP) increased the host survival rate, with 20% of mice surviving to 21 days rather than none when infected with the WT strain. In the tissue culture model, lack of either AcrD or AcrF caused a significant reduction in the ability of *Salmonella* to infect INT 407 cells, with the *acrD* mutant (L132) being more attenuated than the *acrF* mutant (L131). There are several hypotheses to explain these data. First, as inactivation of *acrB* is known to alter the expression of genes found in SPI-1,^35^ it is possible that the inactivation of other RND pump genes also affects the expression of virulence genes. Alternative explanations include the fact that RND efflux pumps export substrates that are required for infection or that the absence of some RND efflux pumps causes damage or stress to the bacterial cell membrane that compromises the ability to cause infection.

The effect of inactivating *acrB* plus one or two other efflux pump genes upon the ability to cause infection was additive. This ability was less attenuated in the *acrDF* (L1395) mutant than in either of the single mutants (L131 and L132). One explanation for this is that *acrB*, *mdtB* and *mdsB* are all overexpressed in this mutant so are able to partially compensate for the functions of the other two systems. The triple mutant lacking AcrB, AcrD and AcrF was unable to adhere to or invade the INT 407 cells. This could suggest that no other transporter could compensate for the loss of these proteins or that the inactivation of multiple RND efflux pump genes causes greater changes in the expression of virulence genes.

The role of efflux pumps in antibiotic resistance makes them targets for the design of inhibitors. Due to the role of efflux pumps in virulence, we also postulate that efflux inhibitors will inhibit virulence as well as augment the activity of antibacterial drugs. Our data show that inhibitors designed to inhibit all RND efflux systems will have a greater antivirulence effect on the organism.

The compensatory expression of efflux pump genes was associated with changes in regulatory gene expression. We hypothesize that the bacterial cell is attempting to increase the expression of the inactivated/deleted genes by increasing the expression of factors known to regulate the expression of RND efflux pump genes such as *ramA*, *marA*, *soxS* and *rob.*^22–24,26,27,43,50^ Our data suggest that these regulators are involved in the modulation of RND efflux pump expression in the absence of homologous systems. The expression of *ramA* was increased when *acrB* was inactivated;^51^ however, the expression of *soxS* was increased when two or more *acr* genes were inactivated. SoxS is also a transcription factor of the AraC/XylS family involved in regulating the response to oxidative stress and genes including *acrAB* and *micF*.^52^ An increased expression of *soxS* could suggest the that lack of efflux accounted for by Acr pump proteins leads to the accumulation of toxic metabolites, as proposed by Rosner and Martin^44,45^ when *E. coli tolC* is inactivated.

The critical role of RND systems in both antimicrobial resistance and the virulence of pathogenic bacteria makes them attractive targets for the design of inhibitors. These molecules could be used to resensitize the bacterium to antimicrobials while simultaneously attenuating the virulence of the infecting organism. Critically, our data indicate that care should be taken when developing efflux pump inhibitors against the RND pumps to determine which pumps are inhibited and to understand the effect of this on the expression of homologous systems. In terms of attenuating virulence, the effect of inhibition was additive so the inhibition of multiple pumps is a good strategy. However, the benefit of this strategy on increasing susceptibility to antimicrobials may be more complex and the impact of this will depend on which drugs are used to treat infections caused by a particular pathogen. For some antimicrobials, an inhibitor with activity against multiple pumps will have a greater impact on susceptibility but an unintended consequence of this may be decreased susceptibility to other drugs, such as the aminoglycosides.

## Funding

This work was funded by an MRC Programme grant (G0501415) to L. J. V. P. and a research grant (GA2011-04R) from BSAC to J. M. A. B. We thank Mark Webber, Michelle Buckner, Lee Rosner and Bill Shafer for reading and constructive criticism of this manuscript prior to submission.

## Transparency declarations

None to declare.

## Supplementary data

Tables S1 and S2 are available as Supplementary data at *JAC* Online (http://jac.oxfordjournals.org/).

Supplementary Data
