# Supplementary material for: Expression of homologous RND efflux pump genes is dependent upon AcrB expression: implications for efflux and virulence inhibitor design
Source: J Antimicrob Chemother. 2014 Oct 6;70(2):424–31. doi: 10.1093/jac/dku380 (PMC4291234; doi:10.1093/jac/dku380)
Supplement: Supplementary Data [file supp_70_2_424__index.html]

Expression of homologous RND efflux pump genes is dependent upon AcrB expression: implications for efflux and virulence inhibitor design — Supplementary Data 

# Expression of homologous RND efflux pump genes is dependent upon AcrB expression: implications for efflux and virulence inhibitor design

## Supplementary Data

Supplementary Data

**Files in this Data Supplement:**

- Supplementary Data - Docx file
