# Supplementary material for: Expression of homologous RND efflux pump genes is dependent upon AcrB expression: implications for efflux and virulence inhibitor design
Source: J Antimicrob Chemother. 2014 Oct 6;70(2):424–31. doi: 10.1093/jac/dku380 (PMC4291234; doi:10.1093/jac/dku380)
Supplement: Supplementary Data [file supp_dku380_dku380supp.docx]

**Supplementary data**

**Table S1.** Primers used in this study.

| Primer | Sequence (5’-3’) |
| --- | --- |
| 16s (*rrsH*) real time RT-PCR F | TACCTGGTCTTGACAT |
| 16s (*rrsH*) real time RT-PCR R | GACTTAACCCAACATTTC |
| *acrB* real time RT-PCR F | GTCCTCAAGTAGCTTCCT |
| *acrB* real time RT-PCR R | GTAATCCGAAATATCCTCCTG |
| *acrD* real time RT-PCR F | CCTTTGTCAAAGCCTCGATTATC |
| *acrD* real time RT-PCR R | CAGGAACAGATACATCACCAG |
| *acrF* real time RT-PCR F | GTTCCTGTCGTGTTGCTA |
| *acrF* real time RT-PCR R | CAAACATCGTCAGGGTATTG |
| *mdtB* real time RT-PCR F | TATCGGCTATCGCTTCCT |
| *mdtB* real time RT-PCR R | TAGAGCGTAACAACCTGAAT |
| *mdsB* real time RT-PCR F | CGATATGTTGATGGTGGTT |
| *mdsB* real time RT-PCR R | GATGGCGAAGTTAGACAG |

**Table S2.** Data from Biolog Phenotype Microarray. In the PM system growth in the presence of four increasing concentrations of each compound is assessed over time by measuring levels of tetrazolium violet. Values shown are arbitrary units calculated from area under the curve of kinetic data and represent the growth in the presence of the stated compound relative to wild type (SL1344). Positive values indicate better growth than SL1344 while negative values show growth at lower levels than SL1344.

|  | Concentration | L110  *acrB::aph* | L132  *acrD::aph* | L131  *acrF::aph* |
| --- | --- | --- | --- | --- |
| **β**-**lactams** |  |  |  |  |
| Cloxacillin | 1 | 0 | 27803 | 28324 |
|  | 2 | 1024 | 26470 | 27987 |
|  | 3 | 0 | 18975 | 21891 |
|  | 4 | 2 | 4 | 14 |
| Nafcillin | 1 | 0 | 25992 | 26970 |
|  | 2 | 0 | 25493 | 26891 |
|  | 3 | 5 | 19521 | 22038 |
|  | 4 | 577 | 695 | 1441 |
| Oxacillin | 1 | 782 | 30383 | 28377 |
|  | 2 | 6 | 23678 | 26177 |
|  | 3 | 161 | 13985 | 12617 |
|  | 4 | 116 | 4 | 705 |
| Phenethicillin | 1 | -291 | 30523 | 29907 |
|  | 2 | -25 | 22862 | 23731 |
|  | 3 | -37 | 6312 | 4986 |
|  | 4 | -33 | 16 | 304 |
| **Tetracyclines** |  |  |  |  |
| Demeclocyline | 1 | -959 | 5106 | 5439 |
|  | 2 | 96 | 11612 | 14632 |
|  | 3 | 353 | 15433 | 18013 |
|  | 4 | 873 | 10173 | 11746 |
| Tetracycline | 1 | 1040 | -2552 | -1879 |
|  | 2 | -13 | -2424 | -671 |
|  | 3 | -3546 | 6133 | 2623 |
|  | 4 | -4332 | 10916 | 6501 |
| Penimepicycline | 1 | 1450 | -1474 | -299 |
|  | 2 | -3796 | 4487 | 3667 |
|  | 3 | -6713 | 14072 | 8493 |
|  | 4 | -1607 | 10552 | 7104 |
| Doxycycline | 1 | 635 | -1165 | -1382 |
|  | 2 | -4053 | -755 | -772 |
|  | 3 | -11302 | 4542 | 6391 |
|  | 4 | -847 | 11287 | 12451 |
| **Macrolides** |  |  |  |  |
| Erythromycin | 1 | -131 | 27448 | 28079 |
|  | 2 | 370 | 25597 | 27177 |
|  | 3 | -16 | 23260 | 22526 |
|  | 4 | -18 | 3888 | 21060 |
| Tylosin | 1 | 2318 | 15019 | 14845 |
|  | 2 | -255 | 26868 | 26779 |
|  | 3 | -409 | 25200 | 24974 |
|  | 4 | -2958 | 565 | -621 |
| Oleandomycin | 1 | -7 | 27532 | 27966 |
|  | 2 | 16 | 26528 | 26711 |
|  | 3 | -6 | 17165 | 19697 |
|  | 4 | 10 | 113 | 146 |
| Josamycin | 1 | -454 | 28413 | 30790 |
|  | 2 | 157 | 29808 | 31153 |
|  | 3 | 112 | 26382 | 26576 |
|  | 4 | 218 | -516 | 3675 |
| Troleandomycin | 1 | -156 | 28209 | 27356 |
|  | 2 | -50 | 26864 | 24979 |
|  | 3 | -53 | 21134 | 20838 |
|  | 4 | -2748 | 18938 | 15753 |
| **DNA topoisomerase inhibitors** | | | | |
| Lomefloxacin | 1 | 725 | -2351 | -1317 |
|  | 2 | 0 | 25355 | 26422 |
|  | 3 | 446 | 8196 | 15328 |
|  | 4 | 1215 | 1973 | 793 |
| Nalidixic Acid | 1 | -564 | 8470 | 9690 |
|  | 2 | 0 | 18449 | 18515 |
|  | 3 | 1 | 10124 | 11347 |
|  | 4 | -14 | -15 | -5 |
| Ofloxacin | 1 | 1 | 27934 | 27044 |
|  | 2 | -25 | 4485 | 6654 |
|  | 3 | -46 | 373 | -46 |
|  | 4 | -4819 | -3024 | -4779 |
| Norfloxacin | 1 | -964 | -2561 | -4126 |
|  | 2 | -1066 | -396 | -418 |
|  | 3 | 0 | 24347 | 26789 |
|  | 4 | -13 | 22621 | 2472 |
| Ciprofloxacin | 1 | 1317 | 12802 | 13365 |
|  | 2 | 0 | 18704 | 16834 |
|  | 3 | -71 | -43 | -57 |
|  | 4 | -39 | -29 | -32 |
| Novobiocin | 1 | -6011 | 17245 | 17023 |
|  | 2 | -3246 | 14126 | 14056 |
|  | 3 | -8 | 10624 | 11487 |
|  | 4 | 1280 | 2947 | 3589 |
